# Preoperative tumor embolization prolongs time to recurrence of meningiomas: a retrospective propensity-matched analysis

**DOI:** 10.1136/neurintsurg-2022-019080

**Published:** 2022-07-08

**Authors:** Taisuke Akimoto, Makoto Ohtake, Shigeta Miyake, Ryosuke Suzuki, Yu Iida, Wataru Shimohigoshi, Takefumi Higashijima, Taishi Nakamura, Nobuyuki Shimizu, Takashi Kawasaki, Katumi Sakata, Tetsuya Yamamoto

**Affiliations:** 1 Neurosurgery, Yokohama City University School of Medicine Graduate School of Medicine, Yokohama, Kanagawa, Japan; 2 Neurosurgery, Yokohama City University Medical Center, Yokohama, Kanagawa, Japan

**Keywords:** Complication, Tumor, Embolic

## Abstract

**Background:**

Meningiomas are often embolized preoperatively to reduce intraoperative blood loss and facilitate tumor resection. However, the procedure is controversial and its effects have not yet been reported. We evaluated preoperative embolization for meningiomas and its effect on postoperative outcome and recurrence.

**Methods:**

We retrospectively reviewed the medical records of 186 patients with WHO grade I meningiomas who underwent surgical treatment at our hospital between January 2010 and December 2020. We used propensity score matching to generate embolization and no-embolization groups (42 patients each) to examine embolization effects.

**Results:**

Preoperative embolization was performed in 71 patients (38.2%). In the propensity-matched analysis, the embolization group showed favorable recurrence-free survival (RFS) (mean 49.4 vs 24.1 months; Wilcoxon p=0.049). The embolization group had significantly less intraoperative blood loss (178±203 mL vs 221±165 mL; p=0.009) and shorter operation time (5.6±2.0 hours vs 6.8±2.8 hours; p=0.036). There were no significant differences in Simpson grade IV resection (33.3% vs 28.6%; p=0.637) or overall perioperative complications (21.4% vs 11.9%; p=0.241). Tumor embolization prolonged RFS in a subanalysis of cases who experienced recurrence (n=39) among the overall cases before variable control (mean RFS 33.2 vs 16.0 months; log-rank p=0.003).

**Conclusions:**

After controlling for variables, preoperative embolization for meningioma did not improve the Simpson grade or patient outcomes. However, it might have effects outside of surgical outcomes by prolonging RFS without increasing complications.

**WHAT IS ALREADY KNOWN ON THIS TOPIC**

- The effects of preoperative meningioma embolization on surgical outcomes in patients with WHO grade I intracranial meningiomas are controversial.

**WHAT THIS STUDY ADDS**

- We found no significant difference in perioperative complications, Simpson grade, modified Rankin Scale (mRS) score at last follow-up or mRS score of 0–2 between the two groups, but there was a reduction in intraoperative estimated blood loss and shorter operation time in the embolization group. Preoperative embolization did not increase complications significantly, while it prolonged recurrence-free survival.

**HOW THIS STUDY MIGHT AFFECT RESEARCH, PRACTICE AND/OR POLICY**

- Our results indicate that preoperative embolization may have valuable effects outside of its surgical effects.

## Background

Preoperative embolization for intracranial meningioma is often performed to reduce blood loss during surgery and facilitate resection.^1 2^ A meta-analysis reported that preoperative embolization helps to reduce blood loss and surgical time during meningioma resection.^3^ However, the results of the randomized controlled trial presented by Iacobucci *et al* 4 did not justify the advantages of preoperative embolization in terms of complications and bleeding, although it significantly reduced operation time.^4^ Moreover, Przybylowski *et al* reported that preoperative meningioma embolization did not improve surgical outcomes in patients with WHO grade I intracranial meningiomas.^5^ Additionally, preoperative embolization does not change the time to recurrence for meningiomas.^2^ Because prospective efficacy studies are lacking, it remains controversial whether the risk of preoperative embolization is justified. Previous retrospective cohort studies have focused on intraoperative blood loss, complication rates, and the association of these parameters with the use of different embolic substances.^5–9^ Reports on the association between embolization and tumor recurrence are limited.

A direct comparison of meningiomas is complicated due to the various tumor locations, histology, and extent of the surgery. This study sought to determine the safety and utility of preoperative embolization and its effect on tumor recurrence. We controlled for important variables related to recurrence in patients who underwent resection with or without preoperative embolization to assess the effect of this process on surgical and patient outcomes, as well as on the time to recurrence.

## Methods

### Study design and participants

We retrospectively reviewed all patients who underwent resection of an intracranial meningioma at our institution between January 2010 and December 2020. The following data were collected from patient medical records: sex, age, tumor location, preoperative symptoms, imaging findings (such as calcification and peritumoral edema), extent of resection, surgical outcome, Simpson grade, perioperative complications, time to recurrence, and modified Rankin Scale (mRS) score. Tumor grades followed the WHO classification of tumors of the Central Nervous System Revised fourth edition. Brain invasion cases were classified as grade II even before revision. The location of the tumor was classified as skull base, non-skull base, and supra- or infratentorial. Skull base/infratentorial meningiomas included tumors located in the anterior fossa, middle fossa, cerebellar tentorium, cerebellopontine angle, foramen magnum, and petroclival regions. Non-skull base meningiomas included tumors located in the convexity, parasagittal line, falx cerebri, and lateral ventricle. Exclusion criteria were age <18 years, follow-up time <6 months, and stereotactic radiotherapy prior to resection. Indications for meningioma surgery were determined based on patient characteristics, such as tumor diameter >3 cm, tumor growth trend (based on follow-up MRI), patient age <70 years, ability to tolerate general anesthesia and surgery, and patient preference.

### Preoperative embolization procedure

Cases requiring embolization were selected based on diagnostic angiography. All cases underwent diagnostic angiography before embolization. Preoperative embolization was performed only when embolization via the middle meningeal artery or the external carotid artery was feasible. The internal carotid artery branch and pial feeder were not embolized. Microcatheters were inserted into feeder arteries as close to the tumor as possible. Particles (polyvinyl alcohol particles or Embosphere, Nippon Kayaku Co Ltd, Tokyo, Japan) were injected until a decrease in tumor staining was observed. Finally, coil embolization was added to the feeder and complete occlusion was achieved angiographically for the treated feeder. These protocols were performed in accordance with those of a recent study on embolization.^10^

### Outcome measures

Tumor recurrence was observed in patients with or without tumor-related symptoms by performing MRI at 3, 6, and 12 months postoperatively, and every year thereafter. The surgical outcome was evaluated by Simpson grade classification based on surgical records and postoperative MRI results.^11^ In addition, patient outcomes were assessed by time to recurrence and mRS score at last follow-up. For the time to recurrence analysis, the recurrence-free period was defined as the period from the date on which the first image was taken to the date on which recurrence was recognized on follow-up images or when significant growth in the volume of the residual tumor was detected. In cases without recurrence, follow-up was terminated at the date of the last radiological evaluation.

### Statistical analyses

Quantitative data are presented as mean and SD and categorical data as frequencies (percentages). We performed propensity score matching using the nearest neighbor within a caliper coefficient of 0.20, between patients in the preoperative embolization and no-preoperative embolization groups. To estimate propensity scores, a logistic regression model of preoperative embolization was fitted as a function of patient demographics and recurrence risk factors including age, sex, symptomatology, tumor location, imaging findings (calcification, cyst formation, edema around the tumor, T2-weighted imaging hyperintensity), tumor size, and MIB-1 index. For comparisons between groups, Pearson’s χ^2^ (Fisher’s exact test) and Wilcoxon/Kruskal–Wallis tests were performed. Kaplan–Meier analyses, log-rank tests, and generalized Wilcoxon tests were performed to measure the association of preoperative embolization with the time to recurrence. In addition, multivariate Cox proportional hazards models were used to measure the independent factors related to time to recurrence. Statistical significance was set at p<0.05. All statistical analyses were performed using JMP 15 (SAS Institute, Cary, North Carolina, USA).

## Results

### Patient characteristics

Between January 2010 and December 2020, 202 resections of intracranial meningiomas were performed at our medical center. Sixteen cases were excluded based on selection criteria; thus,186 patients were included in the study. Of these 186 patients, 71 (38.2%) underwent preoperative embolization while 115 (61.8%) did not. Because 93.6% of the cases were WHO grade I meningiomas, a propensity-matched chart was generated only for WHO grade I cases. The characteristics of all patients (n=186) and of the propensity-matched patients only (n=84) are shown in table 1. Among all patients, the percentage of asymptomatic cases in the embolization group (n=35, 49.3%) tended to be lower than that of the no-embolization group (n=72, 62.6%, p=0.074). Moreover, the proportion of meningiomas with MRI T2 high-intensity lesions and cystic meningiomas was higher in the tumor embolization group (64.7% vs 34.3%, p=0.016% and 17.7% vs 1.8%, p=0.002, respectively). We controlled for patient- and tumor-related variables including age, sex, proportion of asymptomatic cases, tumor location, maximum tumor diameter, MRI T2 high-intensity, calcification, peritumoral edema, cyst formation, and MIB-1 index (table 1). In WHO grade I cases, the MIB-1 index before matching was 2.3±1.6% in the embolization group and 2.6±3.2% in the no-embolization group. These values improved to 2.2±2.4% and 2.2±2.6%, respectively, after matching. No factors were significantly different between the matched groups. Therefore, a comparison using the two matched groups of 42 people each was justified. In addition, postoperative stereotactic radiosurgery was not performed for any case in these propensity-matched patient groups.

**Table 1:** Patient characteristics

	Total n=186 n (%)	All patients			Propensity-matched WHO grade I patients		
		Preoperative tumor embolization		Univariate	Preoperative tumor embolization		Univariate
		Yes n=71 n (%)	No n=115 n (%)	P value	Yes n=42 n (%)	No n=42 n (%)	P value
Age, mean±SD (years)	62.2±12.5	61.5±12.3	62.6±12.5	0.616	60.1±12.6	59.3±14.0	0.540
Sex, female	126 (67.7)	43 (60.6)	83 (72.2)	0.100	14 (33.3)	11 (26.2)	0.474
WHO grade				0.859			>0.999
I	174 (93.6)	67 (94.3)	107 (93.0)		42 (100.0)	42 (100.0)	
II	8 (4.3)	3 (4.2)	5 (4.4)		0	0	
III	4 (2.2)	1 (1.4)	3 (2.6)		0	0	
Asymptomatic	107 (57.5)	35 (49.3)	72 (62.6)	0.074	20 (47.6)	21 (50)	0.827
Tumor location							
Convexity	29 (15.6)	10 (13.9)	19 (16.7)	0.611	6 (14.2)	5 (11.9)	0.742
Skull base	91 (48.9)	37 (52.1)	54 (47.0)	0.494	20 (47.6)	23 (54.8)	0.513
Infratentorial	32 (17.2)	10 (14.1)	22 (19.1)	0.376	6 (14.3)	8 (19.1)	0.558
Other location	63 (33.9)	25 (34.7)	38 (33.3)	0.845	16 (38.1)	14 (33.3)	0.649
Maximum diameter of tumor, mean±SD (mm)	32.6±12.3	32.1±12.5	32.7±12.3	0.738	38.9±10.1	39.4±12.6	0.890
MRI T2 high intensity	48 (38.4)	11 (64.7)	37 (34.3)	**0.016**	22 (52.4)	20 (47.6)	0.663
Calcification	30 (24.0)	3 (17.7)	27 (25.0)	0.509	5 (11.9)	8 (19.1)	0.366
Peritumoral edema	32 (25.6)	5 (29.4)	27 (25)	0.698	19 (45.2)	18 (42.9)	0.826
Cyst formation	5 (4.0)	3 (17.7)	2 (1.8)	**0.002**	1 (2.4)	1 (2.4)	>0.999
MIB-1 index, mean±SD (%)	2.4±4.3	5.1±1.0*	2.0±0.4*	0.494*	2.2±2.4	2.2±2.6	0.544

*In WHO grade Ⅰ cases, embolization vs no embolization 2.3±1.6% vs 2.6±3.2% (p=0.439).

MRI, magnetic resonance imaging; WHO, World Health Organization.

### Perioperative complications and patient outcome

Surgical outcomes including intraoperatively estimated blood loss, operation time, Simpson grade, and mRS score are shown in table 2. Perioperative complication cases with obvious cerebral hemorrhage or cerebral infarction that caused neurological symptoms were included in cerebral hemorrhage or cerebral infarction cases, rather than neurological complication cases. Intraoperative blood loss was significantly lower (178±203 mL vs 221±165 mL; p=0.009) and operation time was significantly shorter (5.6±2.0 hours vs 6.8±2.8 hours; p=0.036) in the embolization group than in the no-embolization group. Overall, the Simpson grade did not differ significantly between the groups (p=0.185). There were 14 cases (33.3%) of Simpson grade IV in the embolization group compared with 12 cases (28.6%) in the no-embolization group, which was not significantly different.Thank you for pointing that out.

**Table 2:** Surgical outcome in the propensity-matched WHO grade I group

Outcome	Preoperative tumor embolization		Univariate
	Yes=42 n (%)	No=42 n (%)	P value
Intraoperative estimated blood loss, mean±SD (mL)	178±203	221±165	**0.009**
Operation time, mean±SD (hours)	5.6±2.0	6.8±2.8	**0.036**
Simpson grade			0.185
I	7 (16.7)	9 (21.4)	
II	21 (50.0)	17 (40.5)	
III	0	4 (9.5)	
IV	14 (33.3)	12 (28.6)	0.637
Complications			
Embolization			
Neurological deficits	0	0	
Cerebral infarction	2 (4.8)	0	
Cerebral hemorrhage	1 (2.4)	0	
Total	3 (7.1)	0	
Surgery			
Neurological deficits (transient)	3 (7.1)	1 (2.4)	
Neurological deficits (permanent)	2 (4.8)	1 (2.4)	
Cerebral infarction	0	1 (2.4)	
Cerebral hemorrhage	1 (2.4)	2 (4.8)	
Total	6 (14.3)	5 (11.9)	0.746
Combined total complications	9 (21.4)	5 (11.9)	0.241
Postoperative recurrence	5 (11.9)	9 (21.4)	0.241
mRS at last follow-up			0.268
0	25 (59.5)	19 (45.2)	
1	12 (28.6)	11 (26.2)	
2	4 (9.2)	8 (19.1)	
3	0	3 (7.1)	
4	1 (2.4)	1 (2.4)	
5	0	0	
6	0	0	
mRS 0–2 at last follow-up	41 (97.6)	38 (90.5)	0.167
Mean follow-up period (months)	49.1±25.5	44.7±36.5	0.108

mRS, modified Rankin Scale.

Embolization- and surgery-related complications are shown in table 2. Three patients (7.1%) with preoperative embolization had complications related to the embolization procedure: two patients (4.8%) had cerebral infarction and one (2.4%) had an intratumoral hemorrhage after embolization. Of the two stroke cases, one was an embolisate reflux case and the other had an asymptomatic stroke associated with catheterization, which showed high signal diffusion on MRI the day after embolization. The hemorrhage case was treated with emergency tumor resection and the patient did not experience any neurological complications. As shown in table 2, the profile of surgical complications associated with meningioma resection did not differ significantly between the two groups (p=0.746). No significant difference in the total number of embolization- and surgery-related complications was observed between the groups (21.4％ vs 11.9%; p=0.241).

The mean follow-up period for the embolization and no-embolization groups was 5.6±2.0 months and 6.8±2.8 months, respectively (p=0.108). The last follow-up mRS scores did not differ significantly between the groups (p=0.268). Moreover, no significant differences were observed when the final outcome was stratified by favorable (mRS score of 0–2) or unfavorable (mRS score of 3–6) functional status (mRS 0–2 in 97.6% of embolization patients vs 90.5% of no-embolization patients; p=0.167).

### Recurrence-free survival by embolization status

**Figure 1:**
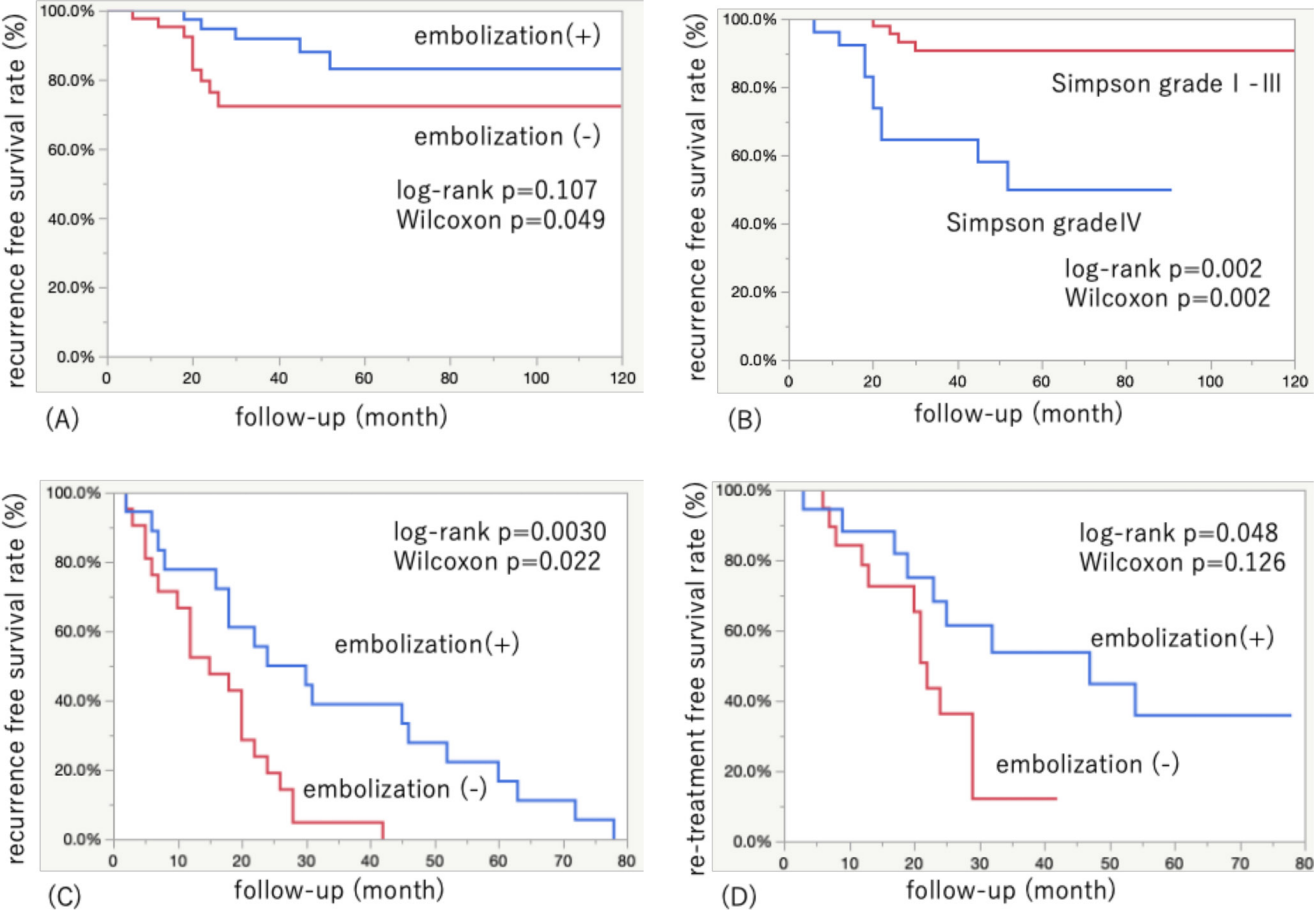
Kaplan–Meier curves of time to recurrence in the propensity-matched analysis of 42 pairs and among all recurrence cases (n=39) of patients with meningioma, with and without preoperative embolization. (A) Recurrence-free survival (RFS) in the preoperative embolization group versus no-embolization group (mean RFS 49.4 vs 24.2 months; log-rank p=0.107; Wilcoxon p=0.049). (B) RFS in patients with Simpson grade I–III versus Simpson grade IV (mean RFS 39.4 vs 29.6 months; log-rank p=0.002; Wilcoxon p=0.002). (C) RFS in preoperative embolization group versus no-embolization group (median RFS 27 vs 15 months; log-rank p=0.0030; Wilcoxon p=0.022). (D) Retreatment-free survival in preoperative embolization group versus no-embolization group (median 47 vs 22 months; log-rank p=0.048; Wilcoxon p=0.126).

Of the 82 patients, 14 (16.7%) experienced tumor recurrence. The median recurrence-free survival (RFS) was 49.3 months in patients with embolization and 24.2 months in patients without embolization. In Kaplan–Meier analysis, the log-rank test showed no significant difference between the embolization and no-embolization groups, but there was a statistically significant difference by the generalized Wilcoxon test. The results suggested that preoperative tumor embolization might reduce tumor recurrence in the early post-treatment period (log-rank p=0.107; Wilcoxon p=0.049; figure 1A). A sensitivity analysis for WHO grade I meningioma matching without adjustment for Simpson grade and MIB-1 (factors unknown before surgery) resulted in similar findings (see online supplemental figure 1). Multivariate analysis using Cox proportional hazards models for patients with WHO grade Ⅰ meningiomas showed that preoperative embolization (HR 0.23, 95% CI 0.07 to 0.86, p=0.022) and Simpson grade IV resection (HR 9.74, 95% CI 1.81 to 52.4, p=0.0018) were significantly associated with time to recurrence. Kaplan–Meier analysis showed that Simpson grade Ⅳ resection was associated with significantly shorter time to recurrence (log-rank p=0.002; Wilcoxon p=0.002; figure 1B)

10.1136/neurintsurg-2022-019080.supp1 Supplementary data

### Time to recurrence among recurrence cases

A subgroup analysis including patients with WHO grade II/III meningiomas was performed to determine whether analysis of only cases with recurrent meningiomas would yield a difference in outcomes. The characteristics of all recurrence cases (n=39) are shown in table 3. No statistically significant differences between this subset of the embolization (n=18) and no-embolization groups (n=21) were observed in terms of age (p=0.877), WHO grade (p=0.888), skull base (p=0.477), MIB-1 index (p=0.650), Simpson grade (p=0.718), postoperative stereotactic radiosurgery (p=0.802), or mRS score 0–2 at last follow-up (p=0.643). In contrast, the maximum tumor diameter was larger in the embolization group than in the no-embolization group (53.8±13.2 mm vs 39.8±14.3 mm; p=0.006) and the percentage of patients with peritumoral edema was also greater in the embolization group (72.2% vs 33.3%; p=0.015). Kaplan–Meier analysis showed that preoperative tumor embolization prolonged the time to recurrence (log-rank p=0.003; Wilcoxon p=0.022; HR 0.25, 95% CI 0.09 to 0.64; p=0.004; figure 1C). Preoperative tumor embolization also prolonged the time to retreatment (log-rank p=0.048; Wilcoxon p=0.126; HR 0.22, 95% CI 0.06 to 0.81; p=0.023; figure 1D).

**Table 3:** Characteristics of patients with postoperative tumor recurrence in all patients

	Total n=39 n (%)	Preoperative tumor embolization		Univariate
		Yes n=18 n (%)	No n=21 n (%)	P value
Age, mean±SD (years)	63.9±12.7	64.4±8.3	63.4±8.3	0.877
Sex, female	15 (38.5)	5 (27.8)	10 (47.6)	0.204
WHO grade				0.888
I	30 (76.9)	14 (77.8)	16 (76.2)	
II	6 (15.4)	3 (16.7)	3 (14.3)	
III	3 (7.7)	1 (5.6)	2 (9.5)	
Asymptomatic	12 (30.7)	4 (22.2)	8 (38.1)	0.284
Tumor location				
Convexity	4 (10.3)	2 (11.1)	2 (9.5)	0.871
Skull base	24 (61.5)	10 (55.6)	14 (66.7)	0.477
Infratentorial	7 (18.0)	1 (5.6)	6 (28.6)	0.06
Other location	10 (25.6)	6 (33.3)	4 (19.1)	0.308
Maximum diameter of tumor, mean±SD (mm)	46.3±13.8	53.8±13.2	39.8±14.3	**0.0063**
MRI T2 high intensity	16 (41.0)	5 (27.8)	11 (52.4)	0.119
Calcification	2 (5.1)	0	2 (9.5)	0.179
Peritumoral edema	20 (51.3)	13 (72.2)	7 (33.3)	**0.015**
Cyst formation	3 (7.7)	1 (5.6)	2 (9.5)	0.643
MIB-1, mean±SD (%)	7.7±9.4	7.7±2.2	7.7±2.1	0.650
Simpson grade				0.718
I	4 (10.3)	1 (5.6)	3 (14.3)	
II	3 (7.7)	1 (5.6)	2 (9.5)	
III	5 (12.8)	3 (16.7)	2 (9.5)	
IV	27 (69.2)	13 (72.2)	14 (66.7)	
Surgery-related complications	1 (2,6)	1 (5.6)	0	0.274
Postoperative stereotactic radiotherapy	16 (41.0)	7 (38.9)	9 (42.9)	0.802
Retreatment at recurrence	21 (53.4)	9 (50)	12 (57.1)	0.656
mRS 0–2 at last follow-up	36 (92.3)	17 (94.4)	19 (90.5)	0.643

mRS, modified Rankin Scale; WHO, World Health Organization.

## Discussion

We performed a retrospective propensity-matched cohort study of patients who underwent resection of intracranial meningiomas with and without preoperative embolization, adjusting for factors known to be associated with recurrence. Our study found no significant difference in perioperative complications, Simpson grade, or mRS at last follow-up between the two groups, but showed a reduction in intraoperative estimated blood loss and shorter operation time in the embolization group. Although some previous reports indicated that preoperative tumor embolization does not change surgical outcomes,^5^ our results were consistent with existing reports^1 2 4^ of decreased blood loss and a shorter operation time. Preoperative embolization was also somewhat effective as a surgical adjuvant and was performed without a significant increase in complications.

To the best of our knowledge, no previous report has examined the surgical outcomes of embolization for intracranial meningiomas after controlling for specific tumor and patient parameters, and revealed that preoperative embolization prolonged time to recurrence. In contrast, Wirsching *et al* 2 reported that preoperative embolization did not change RFS in patients with WHO grade I meningiomas. However, that study did not adjust for various meningioma factors and was therefore subject to significant selection bias. In addition, because preoperative embolization is often employed when the tumor is large or difficult to resect,^1 12^ selection bias is more likely.

We performed preoperative tumor embolization along with feeder vessel occlusion to the maximum extent possible to ensure minimum blood flow to the tumor. Fukushima *et al* reported a favorable effect of dural detachment on long-term tumor control.^13^ The authors explained that dural detachment involving removal of feeding arteries in the dura mater affected tumor control favorably. In addition, a slightly lower recurrence rate, although not statistically significant, was reported when preoperative embolization was performed in cases of Simpson grade IV resection.^14^ Thus, we consider that devascularization with preoperative embolization improved tumor control. Although further studies are warranted, it is worthwhile investigating the tumor control effect of preoperative embolization in cases of difficult resection.

Because of the diversity of meningiomas, recent reports of meningioma outcomes have had differing results regarding comparisons based on propensity score matching.^5 12^ However, knowing which factors to adjust is vital. We adjusted for factors related to recurrence and tumor growth including age, size, tumor location, and MIB-1 index within WHO grade I meningiomas.^15–17^ These factors were identified by Kaplan–Meier analysis and log-rank tests to be associated with recurrence in our 186 cases (sex, symptoms, MIB-1 index, Simpson grade, tumor size, calcification, and peritumoral edema). Although Simpson grade was related to recurrence, it should be considered with respect to improvement in removal with embolization, and was thus not used as a matching factor. In our study, no significant difference was observed in Simpson grade between the embolization and no-embolization groups. Thus, the bias of the effect of recurrence on the difference in Simpson grade could be eliminated.

In this cohort, the incidence of complications related to embolization was equal to or lower than that reported in previous studies,^1 18^ which indicated that embolization can be safely performed. In tumor embolization, target vessels other than the external carotid artery and use of liquid material have been reported to be associated with the risk of complications.^18^ In this study, embolization was limited to external carotid arteries and the embolization material was non-liquid, which may have contributed to reduced complications.

Our institution aims to reduce complications and avoid neurological morbidity rather than perform overly aggressive resection to raise the Simpson grade. This concept is reflected in the relatively high resection rate of Simpson grade IV during the study period (31.0%), which is likely because about half of the cohort had meningiomas at the skull base. Przybylowski *et al* 19 reported that 118 (39.1%) of 302 skull base cases were resected at Simpson grade IV. Thus, the resection rate did not differ significantly from recent reports. In our study, Simpson grade IV was also an independent risk factor for recurrence, suggesting that the goal of treatment for meningiomas should be to achieve Simpson grade I. Nevertheless, tumor embolization did not improve resectability. Embolization poses certain risks of complications^5 20 21^ and high costs.^22^ However, we believe that the benefit of prolonging the time to recurrence may outweigh the potential risks and cost of embolization.

Tumor embolization is a treatment option for some cancers.^23^ In fact, in hepatocellular carcinoma, embolization plays a major role in the treatment of patients who are not candidates for surgery.^23–25^ However, while tumor embolization prolongs survival, hypoxia after embolization limits the long-term efficacy of this treatment strategy.^25 26^ Hypoxia can further activate angiogenesis and tumor growth, often leading to tumor recurrence, which is a significant factor limiting the therapeutic efficacy of tumor embolization.^27 28^ Furthermore, molecular markers of hypoxia are predictors of meningioma (including WHO grade I) recurrence and growth.^29^ In our study, Kaplan–Meier analysis showed a significant difference in time to recurrence with generalized Wilcoxon tests, but not with log-rank tests, indicating that the inhibitory effect of preoperative embolization on recurrence was prevalent in the short term after embolization and waned in the long term. In the short term, tumor embolization might result in apoptosis and decreased tumor growth (a direct effect) while, in the long term, it may have paradoxical effects such as malignant transformation due to tumor hypoxia. In other words, the molecular biological effects of tumor embolization might depend on the length of time after embolization, and further investigation is needed to elucidate this time-dependent phenomenon.

We performed a subanalysis restricted to recurrent cases because WHO grade I meningiomas alone have a low recurrence rate. In fact, the recurrence rate in this study was 17% (30/174). Thus, we evaluated the effect of embolization in the subgroup of patients who experienced recurrence, including cases of WHO grade II and III, to observe the effect of embolization in preventing recurrence. Although the total number of cases was not large (n=39), there was a clear difference between the two groups, and time to recurrence and time to retreatment were prolonged in the embolization group, even though they typically had larger tumors. This result may indicate that embolization is effective in cases of recurrence. Our data showed no difference in the MIB-1 index between the embolized and non-embolized groups, and no significant difference in the Simpson grade. Previous reports have suggested that embolization may have a negative impact on WHO grade II/III tumors.^2^ This may be because tumor embolization is more likely to be selected in cases with large tumors, abundant blood flow, and in whom it is difficult to remove the tumor, making it prone to bias. Another possibility is that incomplete embolization may cause hypoxia and malignant transformation of the tumor. Both of these possibilities are beyond the realm of prediction, and further case accumulation and investigation of tumor embolization in malignant meningiomas are needed.

This study has several limitations. First, it was a single-center retrospective analysis; however, long-term outcomes were confirmed for almost all cases. This study was also limited by selection bias because we only performed embolization when we believed that it could be performed safely. Furthermore, the calculation of blood loss is often extremely subjective in the operative records, which may bias the results. Of all cases, blood transfusion was only required in two cases and could not be examined. Stereotactic radiosurgery may be added in cases of poor postoperative grade and Simpson grade IV, and subsequent outcomes for grade II/III meningiomas are not directly comparable. For WHO grade I cases, stereotactic radiosurgery was sometimes added in cases of recurrence, but no intervention was performed until recurrence; therefore, it did not influence the present study. By including the MIB-1 index, a factor that cannot be predicted preoperatively, and by using propensity score matching, preoperative embolization was found to increase the time to recurrence significantly. However, we were unable to predict which patients should be embolized preoperatively. We did not investigate differences by molecular classification due to the lack of information on methylation, molecular markers, and genetic mutations. Thus, the short-term prolongation of RFS revealed by the difference between Wilcoxon and log-rank test results needs to be clarified in further studies, including molecular biological evaluation. Finally, although we studied a group of patients who underwent preoperative embolization using a single standardized method, various methods of embolization are available. We also did not study the degree of embolization; in fact, cerebral angiography showed about 60–100% resolution of tumor blush, but we determined that the degree of embolization was heterogeneous and difficult to quantify correctly. From the results of this study, we believe that embolization, at least to the extent that it reduces both intraoperative blood loss and operative time, has the potential to prolong the time to recurrence. Thus, it is necessary to search for the best embolization method from the perspective of recurrence.

## Conclusions

In this study we investigated the tumor inhibitory effect of preoperative tumor embolization on recurrence of meningioma by adjusting for factors related to recurrence. We found that preoperative tumor embolization might prolong the time to recurrence in meningiomas without a significant increase in complications and reduced intraoperative blood loss and operation time, although not sufficiently to improve Simpson grade. Thus, our results imply that preoperative embolization of meningiomas may have an effect beyond its surgical impact.

10.1136/neurintsurg-2022-019080.supp2 Supplementary data

## Data Availability

Data are available upon reasonable request. A synopsis of our original dataset is presented in the current paper. However, additional data including explanatory material and complete datasets are available to fellow researchers on request.
