# Supplementary material for: Preoperative tumor embolization prolongs time to recurrence of meningiomas: a retrospective propensity-matched analysis
Source: J Neurointerv Surg. 2022 Jul 8;15(8):814–20. doi: 10.1136/neurintsurg-2022-019080 (PMC10359541; doi:10.1136/neurintsurg-2022-019080)
Supplement: Supplementary data [file neurintsurg-2022-019080supp001.pdf]

Supplemental figure 1

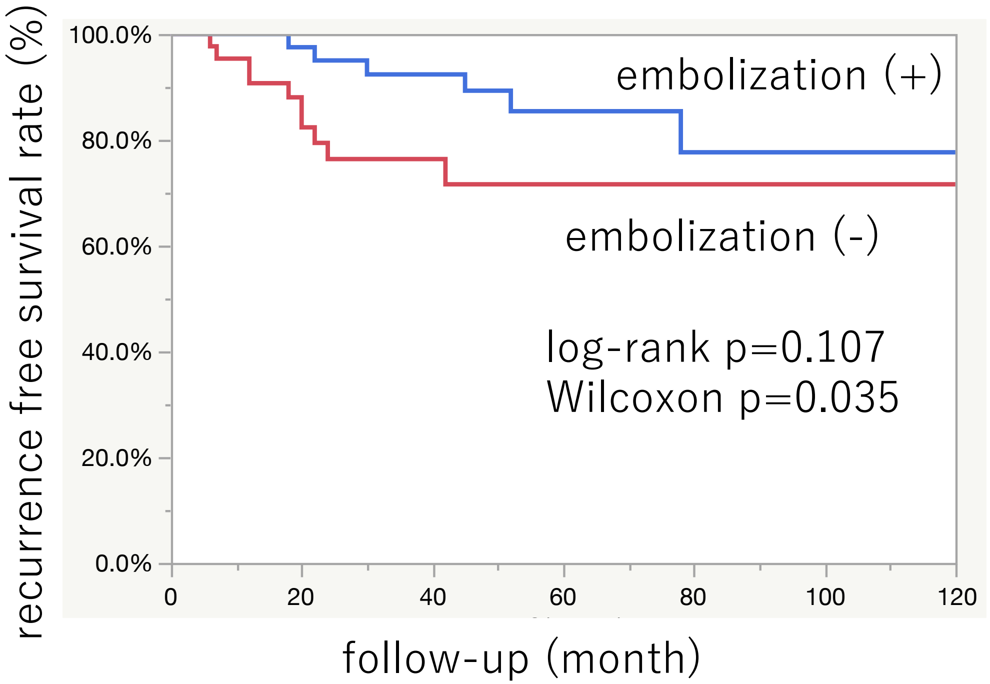

Supplemental Fig. 1 Sensitivity analysis: Kaplan–Meier curves of time to recurrence in the propensity-matched analysis of 43 pairs
We conducted a sensitivity analysis for WHO grade 1 meningioma matching without adjustment for Simpson grade and mib-1 (Factors unknown before surgery).
Recurrence-free survival (RFS) in the preoperative embolization group vs. no-embolization group (mean RFS, 57.2 vs. 41.4 months; log-rank p = 0.107; Wilcoxon p = 0.035).
